# The role of Ca^2+^ influx in endocytic vacuole formation in pancreatic acinar cells

**DOI:** 10.1042/BJ20140398

**Published:** 2015-01-22

**Authors:** Svetlana Voronina, David Collier, Michael Chvanov, Ben Middlehurst, Alison J. Beckett, Ian A. Prior, David N. Criddle, Malcolm Begg, Katsuhiko Mikoshiba, Robert Sutton, Alexei V. Tepikin

**Affiliations:** *Department of Cellular and Molecular Physiology, University of Liverpool, Crown Street, Liverpool L69 3BX, U.K.; †Laboratory for Developmental Neurobiology, Riken Brain Science Institute, 2-1 Hirosawa, Wako City, Saitama 351-0198, Japan; ‡Respiratory Therapy Area Unit, Medicines Research Centre, GlaxoSmithKline, Stevenage SG1 2NY, England, U.K.; §NIHR Liverpool Pancreas Biomedical Research Unit, University of Liverpool, Crown Street, Liverpool L69 3BX, U.K.

**Keywords:** acute pancreatitis, calpain, GSK-7975A, store-operated Ca^2+^ entry, stromal interaction molecule, [Ca^2+^]_c_, cytosolic Ca^2+^ concentration, CCK, cholecystokinin-8, ER, endoplasmic reticulum, fura 2/AM, fura 2 acetoxymethyl ester, SOCE, store-operated Ca^2+^ entry, STIM, stromal interaction molecule, TG, thapsigargin, TLC-S, taurolithocholic acid 3-sulfate

## Abstract

The inducers of acute pancreatitis trigger a prolonged increase in the cytosolic Ca^2+^ concentration ([Ca^2+^]_c_), which is responsible for the damage to and eventual death of pancreatic acinar cells. Vacuolization is an important indicator of pancreatic acinar cell damage. Furthermore, activation of trypsinogen occurs in the endocytic vacuoles; therefore the vacuoles can be considered as ‘initiating’ organelles in the development of the cell injury. In the present study, we investigated the relationship between the formation of endocytic vacuoles and Ca^2+^ influx developed in response to the inducers of acute pancreatitis [bile acid taurolithocholic acid 3-sulfate (TLC-S) and supramaximal concentration of cholecystokinin-8 (CCK)]. We found that the inhibitor of STIM (stromal interaction molecule)/Orai channels, GSK-7975A, effectively suppressed both the Ca^2+^ influx (stimulated by inducers of pancreatitis) and the formation of endocytic vacuoles. Cell death induced by TLC-S or CCK was also inhibited by GSK-7975A. We documented the formation of endocytic vacuoles in response to store-operated Ca^2+^ entry (SOCE) induced by thapsigargin [TG; inhibitor of sarcoplasmic/endoplasmic reticulum (ER) Ca^2+^ pumps] and observed strong inhibition of TG-induced vacuole formation by GSK-7975A. Finally, we found that structurally-unrelated inhibitors of calpain suppress formation of endocytic vacuoles, suggesting that this Ca^2+^-dependent protease is a mediator between Ca^2+^ elevation and endocytic vacuole formation.

## Abstract 2

Endocytic vacuoles are ‘initiating’ organelles in the development of acute pancreatitis. In the present study, we identified the important roles of store-operated Ca^2+^ influx and Ca^2+^-dependent proteases (calpains) in the formation of these organelles.

## INTRODUCTION

Acute pancreatitis is a frequent disease with considerable human and financial costs [1,2]. Vacuolization is a hallmark of acute pancreatitis [3–7]. In our previous study, we observed the formation of large endocytic vacuoles and documented trypsinogen activation in these vacuoles, suggesting an important role for these organelles in the initiation of the disease [7].

Inducers of acute pancreatitis (bile acids, non-oxidative ethanol metabolites and supramaximal doses of secretagogues) trigger characteristic Ca^2+^ responses in pancreatic acinar cells [8–14]. These responses are composed of the initial peak (mainly due to the release of Ca^2+^ from the intracellular stores) and prolonged Ca^2+^ plateau [reflecting the balance of Ca^2+^ influx and Ca^2+^ removal by the plasma membrane Ca^2+^ ATPases (PMCA)] (reviewed in [15]). In non-excitable cells, an important component of Ca^2+^ influx is formed by store-operated Ca^2+^ entry (SOCE). SOCE is activated as a result of the interaction between the stromal interaction molecule [STIM; Ca^2+^ sensor located in the endoplasmic reticulum (ER)] and the plasma membrane-localized channel-forming protein Orai [16–19], reviewed in [20–22]. Other SOCE mechanisms (notably involving transient receptor potential cation channels) were also reported (reviewed in [23]). The recent development by GlaxoSmithKline of a selective inhibitor of Orai channels GSK-7975A [24,25] provided an opportunity to test the contribution of this mechanism to the Ca^2+^ plateau and the formation of the endocytic vacuoles in the pancreatic acinar cells challenged by the inducers of acute pancreatitis. This opportunity was utilized in the current study.

A number of downstream Ca^2+^ effectors could potentially mediate the injurious action of this ion (reviewed in [26,27]). The specific contribution of calpains to the formation of endocytic vacuoles has not been investigated before and formed a part of the current study.

## MATERIALS AND METHODS

### Chemicals

All salts, DMSO, CCK (cholecystokinin-8) and TLC-S (taurolithocholic acid 3-sulfate) were obtained from Sigma Aldrich. Collagenase was from Worthington (Lorne Laboratories). Thapsigargin (TG) was from Calbiochem. Propidium iodide, Lucifer Yellow and fura 2 acetoxymethyl ester (fura 2/AM) were from Life Technologies. All calpain inhibitors were from Calbiochem. PD145305 was from Insight Biotechnology. GSK-7975A was a gift from GlaxoSmithKline.

### Solutions

A standard extracellular solution was used for cell isolation, measurements of vacuole formation and fura 2 loading containing: NaCl (140 mM); KCl (4.7 mM); MgCl_2_ (1.13 mM); CaCl_2_ (1 mM); D-glucose (10 mM); HEPES (10 mM). The solution was adjusted to pH 7.3 using NaOH.

### Animals and cell isolation

All animal experiments were conducted in accordance with the Animals (Scientific Procedure) Act of 1986. Pancreatic acinar cells were isolated from the pancreata of CD1 mice using collagenase digestion, as described previously [28].

### Ca^2+^ and Ba^2+^ imaging

**Figure 1:**
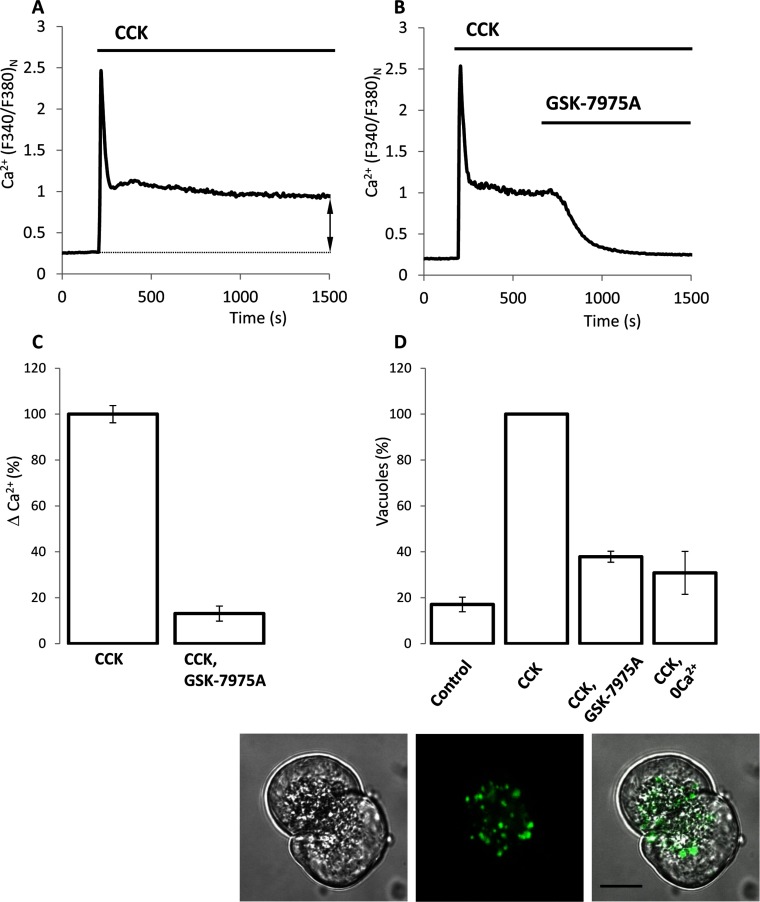
Effect of GSK-7975A on CCK-induced Ca^2+^ plateau and CCK-induced endocytic vacuole formation (**A**) An example trace of the CCK-induced Ca^2+^ response; here and in other Figures it is shown as a normalized ratio of fura 2 fluorescence recorded at excitation with 340 nm and 380 nm. The summary data for this type of experiments is shown in (**C**). The dashed line and double arrow illustrate how the amplitude of ‘Ca^2+^ plateau’ was analysed in this and other experiments shown in Figures 1–4. In experiments shown in (**A**–**C**), [Ca^2+^] in the extracellular solution was 5 mM. (**B**) Inhibition of the plateau component of the CCK-induced Ca^2+^response by GSK-7975A. (**C**) The histogram summarizes the results of experiments illustrated in (**A**) and (**B**) and shows the normalized amplitudes of Ca^2+^ plateau (ΔCa^2+^, calculated as the difference of *F*_340_:*F*_380_ ratio at the end of experiment and just before the addition of CCK) in cells treated with CCK (100±4%, 120 cells) and in cells treated with CCK and GSK-7975A (13±4%, 163 cells). The ΔCa^2+^ in the presence and absence of GSK-7975A were significantly different. (**D**) Effect of GSK-7975A on the formation of endocytic vacuoles. In ‘CCK, 0Ca^2+^’ experiments, CaCl_2_ was not included in the extracellular solution; in all other types of experiments Ca^2+^ concentration in the extracellular solution was 1 mM. The histogram in the upper part of the Figure shows the numbers of vacuoles per cell, normalized to that formed as a result of CCK stimulation (second bar, seven experiments): 17±3% for ‘control’, seven experiments; 38 ±2%, for ‘CCK, GSK-7975A’, four experiments; and 31±9% for ‘CCK, 0Ca^2+^’, three experiments. Approximately 30 cells were analysed in each experiment. The numbers of vacuoles formed in CCK-stimulated cells in the presence and absence of GSK-7975A were significantly different. Lower panel of (**D**) shows the vacuoles formed in pancreatic acinar cells. The Figure is composed of a transmitted light non-confocal image of the acinar cell doublet (left part), fluorescence confocal image of the cells revealing Lucifer Yellow accumulated in vacuoles (central part) and overlay of the two images (right part). Scale bar, 10 μm.

**Figure 2:**
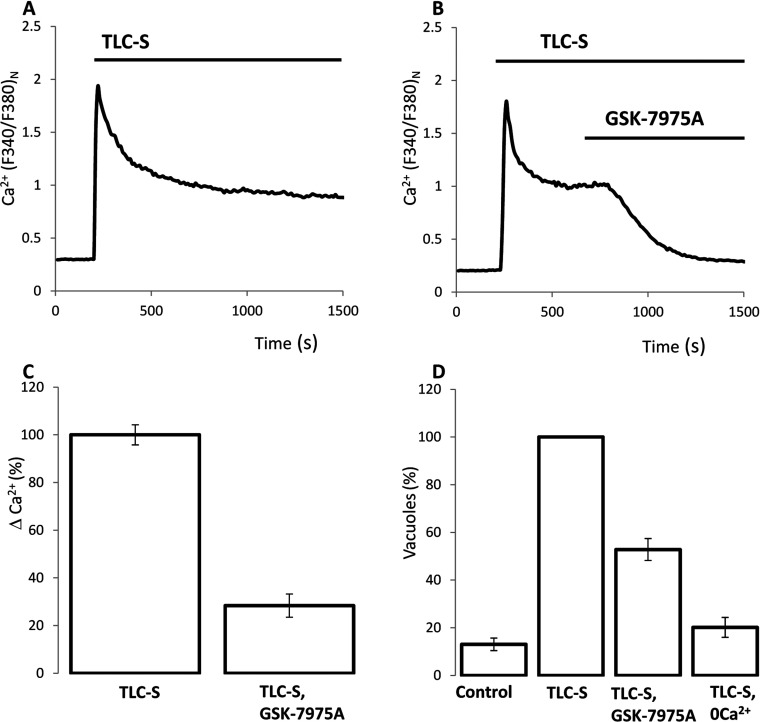
Effect of GSK-7975A on TLC-S-induced [Ca^2+^]_c_ plateau and on TLC-S-induced endocytic vacuole formation (**A**) An example trace of TLC-S-induced [Ca^2+^]_c_ response. In experiments shown in (**A**–**C**) Ca^2+^ in the extracellular solution was 5 mM. (**B**) Inhibition of the plateau component of the TLC-S-induced response by GSK-7975A. (**C**) The histogram summarizes the results of experiments illustrated in (**A** and **B**) and shows the normalized amplitudes of the Ca^2+^ plateau (ΔCa^2+^, calculated as the difference of *F*_340_:*F*_380_ ratio at the end of the experiment and just before the addition of TLC-S) in cells treated with TLC-S (100±5%, 29 cells) and in cells treated with TLC-S and GSK-7975A (28±5%, 28 cells). The ΔCa^2+^ in the presence and absence of GSK-7975A were significantly different. (**D**) Effect of GSK-7975A on the formation of endocytic vacuoles. In ‘TLC-S, 0Ca^2+^’ experiments CaCl_2_ was not included in the extracellular solution; in all other types of experiments [Ca^2+^] in the extracellular solution was 1 mM. The histogram shows the numbers of vacuoles per cell, normalized to that formed as a result of TLC-S stimulation (second bar, seven experiments): 13±3% for ‘control’, seven experiments; 53±5%, for ‘TLC-S, GSK-7975A’, six experiments; and 20±4% for ‘TLC-S, 0Ca^2+^’, three experiments. Approximately 30 cells were analysed in each experiment. The numbers of vacuoles formed in TLC-S-stimulated cells in the presence and absence of GSK-7975A were significantly different.

**Figure 3:**
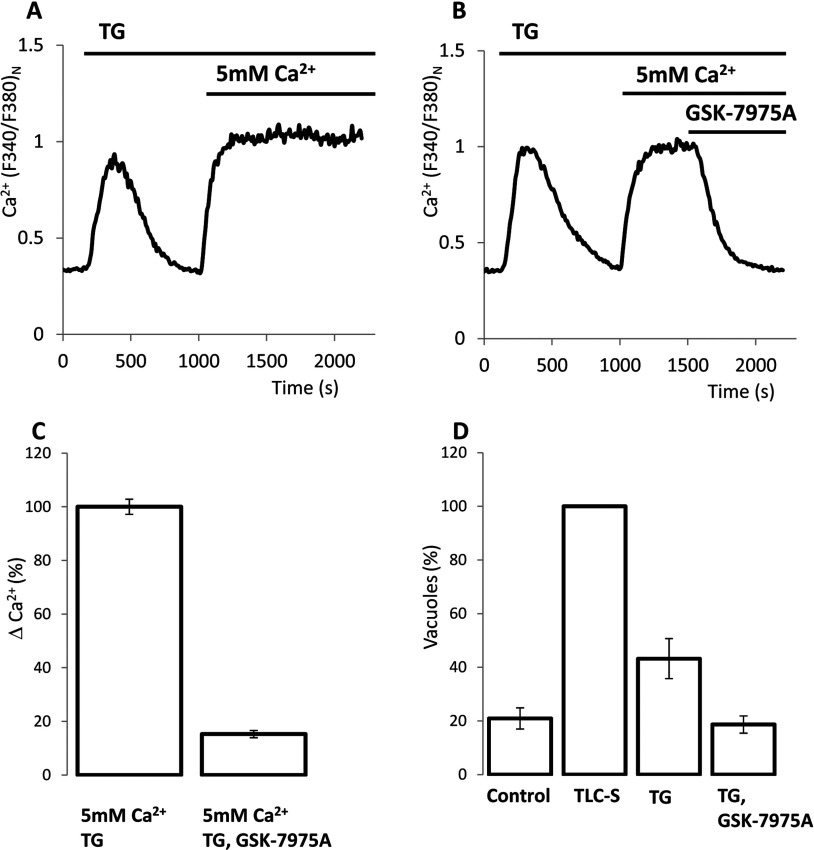
Effect of GSK-7975A on TG-induced [Ca^2+^]_c_ plateau and on the TG-induced formation of endocytic vacuoles (**A**) An example trace of TG-induced [Ca^2+^]_c_ response. In experiments shown in (**A**–**C**) [Ca^2+^] in the extracellular solution was 5 mM. (**B**) Inhibition of the plateau component of the TG-induced response by GSK-7975A. (**C**) The histogram summarizes the results of experiments illustrated in (**A**) and (**B**) and shows the amplitudes of Ca^2+^ plateau (ΔCa^2+^, calculated as the difference of *F*_340_:*F*_380_ ratio at the end of the experiment and just before the addition of TG) in cells treated with TG (100±3%, 161 cells) and in cells treated with TG and GSK-7975A (15±1%, 147 cells). The ΔCa^2+^ in the presence and absence of GSK-7975A were significantly different. (**D**) Effect of GSK-7975A on the TG-induced formation of endocytic vacuoles. For each experiment, the number of vacuoles per cell formed as a result of TG treatment (with or without GSK-7975A) was normalized to that formed as a result of TLC-S stimulation (second bar) in the cell preparation from the same animal. Such normalization allowed us to reduce variability and to compare the TG response with the maximal vacuolization achievable in the individual cell preparations. The histogram shows the numbers of vacuoles per cell, normalized to that formed as a result of TLC-S stimulation (second bar, five experiments): 21±4% for ‘control’, three experiments; 43±7%, for ‘TG’, five experiments; and 19±3% for ‘TG, GSK-7975A’, four experiments; Approximately 30 cells were analysed in each experiment. The numbers of vacuoles formed in TG-stimulated cells in the presence and absence of GSK-7975A were significantly different.

**Figure 4:**
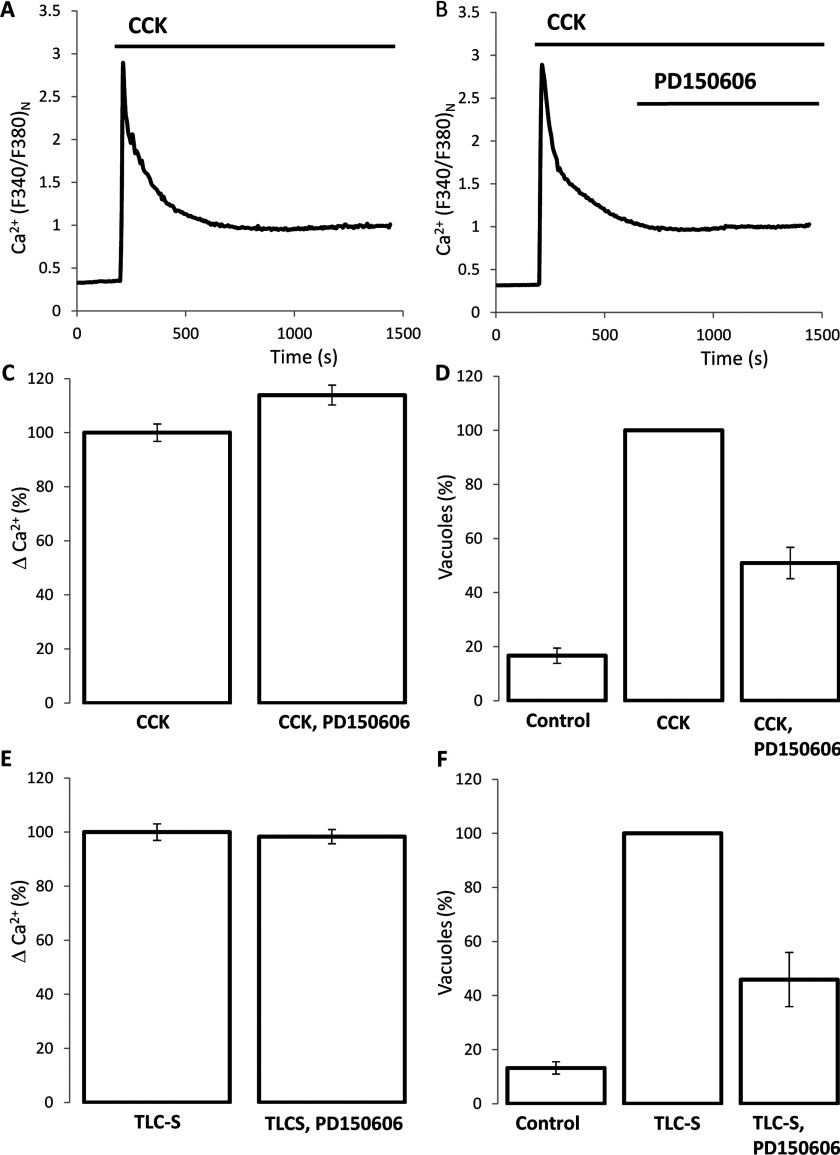
Effect of the calpain inhibitor PD150606 on the formation of endocytic vacuoles (**A**) An example trace of CCK-induced [Ca^2+^]_c_ response. In experiments shown in (**A–C**) and (**E**), Ca^2+^ concentration in the extracellular solution was 5 mM. (**B**) Application of PD150606 did not inhibit the plateau component of the CCK-induced [Ca^2+^]_c_ response. (**C**) The histogram summarizes the results of the experiments illustrated in (**A** and **B**) and shows the amplitudes of the normalized Ca^2+^ plateau (ΔCa^2+^, calculated as the difference of *F*_340_:*F*_380_ ratio at the end of the experiment and just before the addition of CCK) in cells treated with CCK (100±3%, 85 cells) and in cells treated with CCK and PD150606 (113±4%, 123 cells). (**D**) Effect of PD150606 on the CCK-induced formation of endocytic vacuoles. The histogram shows the numbers of vacuoles per cell, normalized to that formed as a result of CCK stimulation (second bar, seven experiments): 16±3% for ‘control’, six experiments; 51±6% for ‘CCK, PD150606’, seven experiments. Approximately 30 cells were analysed in each experiment. The numbers of vacuoles formed in CCK-stimulated cells in the presence and absence of PD150606 were significantly different. (**E**) The histogram summarizes the results of experiments involving PD150606 applications to TLC-S-stimulated cells and shows the normalized amplitudes of Ca^2+^ plateau (ΔCa^2+^, calculated as the difference of *F*_340_:*F*_380_ ratio at the end of experiment and just before the addition of TLC-S) in cells treated with TLC-S (100±3%, 92 cells) and in cells treated with TLC-S and PD150606 (98±3%, 97 cells). The ΔCa^2+^ in the presence and absence of PD150606 were not significantly different. (**F**) Effect of PD150606 on the TLC-S-induced formation of endocytic vacuoles. The numbers of vacuoles per cell normalized to that formed as a result of TLC-S stimulation (second bar, three experiments) were: 13±2% for ‘control’ and 46±10% for ‘TLC-S, PD150606’. Each bar of the histogram represents three experiments. Approximately 30 cells were analysed in each experiment. The numbers of vacuoles formed in TLC-S-stimulated cells in the presence and absence of PD150606 were significantly different.

Freshly isolated pancreatic acinar cells were loaded with fura 2 by incubation for 30–40 min with fura 2/AM (5 μM) at room temperature. Cells were then centrifuged to remove any fura 2/AM remaining in the extracellular solution. Pancreatic acinar cells were plated on to a poly-L-lysine-coated coverslip attached to a superfusion chamber. Depending on the type of experiment, CaCl_2_ was added to or not included in the extracellular solution described above. The Ca^2+^ concentration in the superfusion solution is indicated in the description of specific experiments. In the specified experiments, Ca^2+^ in the extracellular solution was substituted by Ba^2+^ which, in a similar way to Ca^2+^, reacts with the fura 2 and is used to study the influx of divalent cations [21]. Cells were imaged using a Till Photonics Imaging system. Fluorescence of cells loaded with fura 2 was excited at 340 nm and 380 nm and emission collected using a 510 nm band-pass filter. Data are expressed in the Figures as the ratio of fluorescence excited by 340 nm (*F*_340_) and 380 nm (*F*_380_), after corresponding background subtraction. In experiments illustrated by Figures 1, 2 and 4, the ratio at 696 s was determined and used to normalize each individual trace. This time-point is just before the addition of SOCE or calpain inhibitors (and the value just before the addition of inhibitors was used for normalization). In experiments involving TG-induced SOCE (Figure 3), the traces were normalized by the ratio values measured at 1500 s, which correspond to measurements taken just before the addition of the GSK-7975A. In experiments involving SOCE measurements, the difference in the ratio *F*_340_:*F*_380_ at the end of the experiment and that before the agonist application was determined and used as the indication of SOCE (or SOCE modified by inhibitions). This difference for control experiments (without inhibitors) in each group was considered as 100%. Each type of experiment was repeated using at least three animals. The number of cells for each type of experiment is indicated in the Results and Discussion sections. In data presentation (for all components of the study), results are means±S.E.M.. The results were analysed using Student's *t* test, *P*<0.05 was considered statistically significant.

### Imaging vacuole formation

Freshly isolated pancreatic acinar cells were plated on to poly-L-lysine-coated glass-bottomed Petri dishes from MatTek. Extracellular solution was removed and quickly replaced with the incubation solution containing membrane-impermeable fluorescent probe Lucifer Yellow (2 mM), plus test compound(s) (e.g. CCK or TLC-S with or without a SOCE inhibitor) as specified in the Figures. Petri dishes were incubated for 1 h at 35°C. Cells were imaged on a Zeiss 510 confocal microscope with water-immersion objective ×63/numerical aperture 1.2; axial resolution was <1 μm. Fluorescence was excited using a 458 nm laser line; the emission was recorded at wavelengths longer than 505 nm. *Z*-stacks of confocal images (approximately 20 stacks/cell) were taken and the number of vacuoles counted. The numbers of individual experiments (at least three for each condition) are indicated in the Results and Discussion sections or in Figure legends. For these experiments, we set aside Petri dishes with attached acinar cells and separately counted vacuoles in unstimulated cells (control) and in cells stimulated by supramaximal concentrations of CCK or TLC-S. The effects of putative inhibitors or inducers of vacuole formation were normalized to the corresponding response induced by supramaximal CCK or TLC-S.

### Cell-death assay

Cell death of pancreatic acinar cells was measured using a propidium iodide necrosis assay. Pancreatic acinar cells isolated from a single mouse pancreas were suspended in 6 ml of standard extracellular solution. Samples of cell-containing solution (340 μl each) were supplemented with propidium iodide (final concentration 10 μg/ml), placed in individual wells of a 96-well plate and maintained for 30 min at 35°C. This is defined as the pre-treatment period. A POLARstar Omega Plate Reader (BMG Labtech) was used to measure the fluorescence changes. Excitation and emission filters were 540 nm and 590 nm LP (long-pass) respectively. Fluorescence was recorded during the last 10 min of the pre-treatment period and the average value was used as *F*_0_ in subsequent normalization *F*/*F*_0_. Then the vehicle control solutions or solutions containing agonists (CCK or TLC-S) with or without inhibitors (GSK-7975A or PD150606) were added to the cell-containing wells (the volume of the added solutions was 39 μl). Cells were maintained at 35°C and the fluorescence recorded at the specified time. An increase in fluorescence reflects cell death.

### Measurements of amylase secretion

Amylase activity was measured with a Phadebas amylase test kit (Magle Life Sciences) according to the manufacturer's instructions. Dispersed mouse pancreatic acinar cells were pre-incubated at room temperature with extracellular solution containing 10 μM GSK-7975A or 10 μM PD150606 or 0.1% DMSO for 30 min. Subsequently, extracellular solution was replaced with CCK-containing solution (supplemented with 10 μM GSK-7975A or 10 μM PD150606 or 0.1% DMSO). Cells were incubated at 35°C for 30 min. Supernatant was collected and tested for amylase activity. Cells were then treated with 1% Triton X-100 and the amylase activity was measured. Total amylase activity was calculated as the sum of the secreted amylase activity and the amylase activity measured after Triton X-100 treatment. The secreted amylase activity was normalized to the total amylase activity.

## RESULTS

### Effect of GSK-7975A on CCK-induced Ca^2+^ influx and the CCK-induced formation of endocytic vacuoles

The cytosolic Ca^2+^ concentration ([Ca^2+^]_c_) response to supramaximal concentration of CCK (1 nM) resulted in a typical peak-plateau type of response (Figure 1A). 10 μM GSK-7975A has been reported to induce maximal inhibition of Ca^2+^ influx in Jurkat T-cells [25]. The selectivity of GSK-7975A against a plethora of receptors, ion channels and signalling molecules was systematically investigated up to and including 10 μM GSK-7975A [25]. In our experiments, application of 10 μM GSK-7975A strongly (by approximately 87%) inhibited the CCK-induced [Ca^2+^]_c_ plateau (Figures 1B and 1C). GSK-7975A at 3 μM had a less-pronounced inhibitory effect on the [Ca^2+^]_c_ plateau (inhibition by 65±2%, 161cells; result not shown). When applied 30 min before CCK stimulation, 10 μM GSK-7975A did not modify the amplitude of the initial CCK-induced [Ca^2+^]_c_ peak; the amplitude, normalized to the peak of CCK response, was 100±9% in control (174 cells; results not shown) and 101±6% in the presence of GSK-7975A (183 cells; results not shown). These experiments indicate that the GSK-7975A is unlikely to modify the ER Ca^2+^ handling (i.e. Ca^2+^ leak from the ER and Ca^2+^ uptake into the ER).

Ba^2+^ is frequently used as a substitute for Ca^2+^ to study influx/current of divalent cations [21]. In our experiments, 10 μM GSK-7975A did not induce any additional Ba^2+^ influx in unstimulated cells (Supplementary Figure S1, 92 cells), suggesting that this compound is unlikely to stimulate Ca^2+^ leak into the cell; GSK-7975A actually inhibited slow Ba^2+^influx in unstimulated pancreatic acinar cells.

CCK-induced formation of endocytic vacuoles (Figure 1D and Supplementary Movie S1). This process was strongly (by approximately 62%) suppressed by 10 μM GSK-7975A, suggesting that prolonged Ca^2+^ influx is important for vacuole formation (Figure 1D). Removal of Ca^2+^ from the extracellular solution also strongly inhibited vacuole formation (Figure 1D). We next tested the reversibility of the GSK-7975A effect. The procedure, involving treatment of the acinar cells with 10 μM GSK-7975A for 30 min followed by the removal of this compound just before CCK application, resulted in the substantial inhibition of the endocytic vacuole formation (Supplementary Figure S2, 67 cells), suggesting that this effect of GSK-7975A is not easily reversible. We were able to partially reverse the inhibitory effect of GSK-7975A on Ca^2+^ influx but this required a very prolonged wash period (Supplementary Figure S3).

### Effect of GSK-7975A on TLC-S-induced Ca^2+^ influx and the TLC-S-induced formation of endocytic vacuoles

Another inducer of acute pancreatitis TLC-S (500 μM) triggered a strong Ca^2+^ release from the intracellular Ca^2+^ stores of pancreatic acinar cells (seen as the initial peak in [Ca^2+^]_c_ recordings; Figure 2A) followed by a prolonged Ca^2+^ influx (manifested as the plateau; Figure 2A). GSK-7975A at 10 μM clearly (by approximately 72%) suppressed the TLC-S-induced Ca^2+^ plateau (Figures 2B and 2C). TLC-S-induced vacuolization was also significantly (by approximately 47%) suppressed by 10 μM GSK-7975A (Figure 2D). Similarly to experiments with CCK, the removal of extracellular Ca^2+^ resulted in the inhibition of TLC-S-induced vacuole formation and the degree of the inhibition was somewhat stronger that that produced by GSK-7975A (Figure 2D). The incomplete inhibition of the TLC-S-induced vacuole formation by GSK-7975A (as well as weaker inhibition than that produced by removal of extracellular Ca^2+^) is consistent with the incomplete inhibition of the TLC-S-induced Ca^2+^ plateau by GSK-7975A.

### GSK-7975A inhibits thapsigargin-induced store-operated Ca^2+^ entry and vacuole formation induced by thapsigargin

The treatment of the acinar cells with the sarcoplasmic/endoplasmic reticulum Ca^2+^-ATPase (SERCA) pump inhibitor TG (1 μM) resulted in a relatively slow Ca^2+^ release from the intracellular stores manifested as a moderate transient [Ca^2+^]_c_ increase observed in Ca^2+^-free extracellular solution (Figure 3A). The [Ca^2+^]_c_ plateau that was formed as a result of Ca^2+^ addition to the extracellular solution reflects SOCE (Figure 3A). The TG-induced [Ca^2+^]_c_ plateau in the acinar cells was very effectively (by approximately 85%) inhibited by GSK-7975A (Figures 3B and 3C). Endocytic vacuoles formed as a result of TG treatment (Figure 3D; Supplementary Figure S4) but the number of vacuoles formed by TG treatment (3.2±0.5 vacuoles/cell, five experiments and 155 cells) was smaller than that produced by TLC-S (8.4±0.8 vacuoles/cell, 16 experiments and 484 cells) or by the supramaximal concentration of CCK (7.1±0.5 vacuoles/cell, 16 experiments and 524 cells), suggesting that the SOCE is not the only mechanism responsible for the vacuole formation and that the initial powerful Ca^2+^ release and/or other second messengers generated by TLC-S or CCK also play an important role. However, TG treatment clearly resulted in the formation of endocytic vacuoles and this increase was efficiently inhibited by GSK-7975A, indicating that SOCE (i.e. SOCE that is not modified by the second messengers produced by CCK or TLC-S addition) is capable of inducing the formation of endocytic vacuoles.

### The role of calpain in the formation of endocytic vacuoles

We next looked at possible downstream effectors that may link the Ca^2+^ rise to vacuole formation. Calpains (Ca^2+^-dependent proteases) were recently reported as the effectors responsible for the vacuolization in the acinar cells [29]. There are a number of different types of vacuoles that form simultaneously by different mechanisms in the acinar cells [7,30]. The role of calpains in the formation of endocytic vacuoles (i.e. in vacuoles responsible for trypsinogen activation) has not been addressed before and was therefore investigated in the present study. The calpain inhibitor, PD150606, did not inhibit the CCK-induced [Ca^2+^]_c_ plateau (Figures 4A–4C) but significantly (by approximately 49%) inhibited the CCK-induced formation of endocytic vacuoles (Figure 4D). The inhibitory effect of PD150606 on vacuole formation was reversible (Supplementary Figure S2). PD150606 did not significantly change basal or stimulated amylase secretion (Supplementary Figure S5; note that mild inhibition of secretion was induced by GSK-7975A).

PD145305 is an inactive analogue of the calpain inhibitor PD150606. The application of PD145305 had no resolvable effect on the vacuole formation when applied at 10 μM (five experiments, results not shown) or 30 μM (five experiments, results not shown). The CCK-induced formation of endocytic vacuoles was also inhibited by the calpain inhibitor II (applied at 100 μM, inhibition by 23±5%, three experiments; result not shown) and calpain inhibitor III (applied at 100 μM, inhibition by 52±7%, five experiments; result not shown). It should be noted, however, that calpain inhibitor III had a small but statistically significant effect on the [Ca^2+^]_c_ plateau (inhibition by 27±2%, 184 cells, results not shown), whereas calpain inhibitor II had no effect on the CCK-induced plateau [Ca^2+^]_c_ (110 cells, results not shown). The similarity between the effect of calpain inhibitors on the CCK-induced formation of endocytic vacuoles suggests that calpain plays an important role in the formation of this type of vacuoles. We then tested the role of calpains in the TLC-S-induced formation of endocytic vacuoles and observed that all calpain inhibitors significantly suppressed vacuole formation. Thus PD150606 at 10 μM concentration significantly suppressed TLC-S-induced vacuole formation (by approximately 54%; Figure 4F) with no resolvable effect on the TLC-S-induced [Ca^2+^]_c_ plateau (Figure 4E). Calpain inhibitor II inhibited vacuole formation by 38±4% (three experiments, results not shown) and did not inhibit the TLC-S-induced [Ca^2+^]_c_ plateau (123 cells, results not shown) and calpain inhibitor III inhibited vacuole formation by 59±8% (three experiments, results not shown). The similarity of the action of different calpain inhibitors suggests that these proteases are important for TLC-S-induced vacuole formation.

The observed inhibition of endocytic vacuole formation by GSK-7975A and by the calpain inhibitors suggests that these compounds could have an overall protective effect against Ca^2+^ toxicity. We have indeed resolved the inhibition of cell death by 10 μM GSK-7975A and by 10 μM PD150606 (Supplementary Figure S6). The inhibition was apparent for cells stimulated by CCK (Supplementary Figure S6A) and for cells stimulated by TLC-S (Supplementary Figure S6B). These findings are consistent with the notion that the formation of endocytic vacuoles is an important early step in the development of cell damage triggered by the inducers of acute pancreatitis.

## DISCUSSION

Endocytic vacuoles can be up to 10 μm in diameter and appear specifically in pathological conditions as a result of aberrant retrieval of structures formed by compound endocytosis [7]. The co-localization hypothesis of trypsinogen activation suggests intracellular/intra-organellar activation of trypsinogen and the formation of active (and potentially damaging) trypsin as a result of fusion of trypsinogen containing organelles with organelles containing lysosomal proteases (reviewed in [31]). In our previous study, we have indeed detected trypsin activity in the endocytic vacuoles and observed that some of the vacuoles have lysosomal markers [7]. In the present study, we specifically addressed the question about the role of SOCE in the formation of these vacuoles. The results of our experiments revealed that the SOCE-mediated Ca^2+^ plateaus produced by CCK or TLC-S (two commonly used inducers of experimental pancreatitis) are effectively suppressed by the Orai1 inhibitor GSK-7975A and that this is accompanied by a significant reduction in the number of endocytic vacuoles. It is worth noting that GSK-7975A did not completely inhibit the Ca^2+^ plateau induced by CCK and did not abolish amylase secretion. Vacuolization was also effectively suppressed by structurally unrelated calpain inhibitors, suggesting that these Ca^2+^-dependent proteases are downstream effectors in the process of Ca^2+^-induced formation of endocytic vacuoles. The calpain inhibitor PD150606, which was the primary focus of our investigation, did not inhibit Ca^2+^ influx or amylase secretion.

The effective suppression of vacuolization by GSK-7975A and PD150606 indicate that these compounds could be useful for reducing cellular and tissue damage in acute pancreatitis and should therefore be further tested in *in vivo* models of this important disease (with the ultimate aim of developing a treatment). The findings that both compounds reduced cell death of CCK- and TLC-S-treated cells but did not abolish a physiological response (amylase secretion) strengthen the case for further *in vivo* testing of these compounds with the aim of development of a treatment. These further investigations are, however, beyond the scope of the present study.

When the present study was in preparation, a paper from Petersen's group from Cardiff was published [32]. In this publication, Gerasimenko et al. [32] did not explore the role of SOCE in vacuolization but have characterized the role of GSK-7975A in trypsinogen activation and necrosis of pancreatic acinar cells (phenomena which are probably downstream from the formation of endocytic vacuoles). The two studies (ours and [32]) used different inducers of acute pancreatitis and probed different manifestations of cellular damage. The results of these studies are, however, complementary, as both found report that SOCE inhibition reduced the magnitude of the cell damage (albeit different manifestations of the damage), suggesting that this mechanism is a potentially important target for the development of treatment against acute pancreatitis. Our study also indicates that SOCE-induced damage is mediated by calpains and therefore suggests an additional avenue for further research efforts aimed at understanding and alleviating acute pancreatitis.

## Online data

Supplementary data

Supplementary Movie S1. Endocytic vacuoles in pancreatic acinar cells

## References

[1] Neoptolemos J. P., Raraty M., Finch M., Sutton R. (1998). Acute pancreatitis: the substantial human and financial costs. Gut.

[2] Yadav D., Lowenfels A. B. (2013). The epidemiology of pancreatitis and pancreatic cancer. Gastroenterology.

[3] Kim M. S., Lee K. P., Yang D. K., Shin D. M., Abramowitz J., Kiyonaka S., Birnbaumer L., Mori Y., Muallem S. (2011). Genetic and pharmacologic inhibition of the Ca^2+^ influx channel TRPC3 protects secretory epithelia from Ca^2+^-dependent toxicity. Gastroenterology.

[4] Otani T., Chepilko S. M., Grendell J. H., Gorelick F. S. (1998). Codistribution of TAP and the granule membrane protein GRAMP-92 in rat caerulein-induced pancreatitis. Am. J. Physiol..

[5] Raraty M., Ward J., Erdemli G., Vaillant C., Neoptolemos J. P., Sutton R., Petersen O. H. (2000). Calcium-dependent enzyme activation and vacuole formation in the apical granular region of pancreatic acinar cells. Proc. Natl. Acad. Sci. U.S.A..

[6] Saluja A., Saito I., Saluja M., Houlihan M. J., Powers R. E., Meldolesi J., Steer M. (1985). *In vivo* rat pancreatic acinar cell-function during supramaximal stimulation with cerulein. Am. J. Physiol..

[7] Sherwood M. W., Prior I. A., Voronina S. G., Barrow S. L., Woodsmith J. D., Gerasimenko O. V., Petersen O. H., Tepikin A. V. (2007). Activation of trypsinogen in large endocytic vacuoles of pancreatic acinar cells. Proc. Natl. Acad. Sci. U.S.A..

[8] Criddle D. N., Raraty M. G., Neoptolemos J. P., Tepikin A. V., Petersen O. H., Sutton R. (2004). Ethanol toxicity in pancreatic acinar cells: mediation by nonoxidative fatty acid metabolites. Proc. Natl. Acad. Sci. U.S.A..

[9] Husain S. Z., Prasad P., Grant W. M., Kolodecik T. R., Nathanson M. H., Gorelick F. S. (2005). The ryanodine receptor mediates early zymogen activation in pancreatitis. Proc. Natl. Acad. Sci. U.S.A..

[10] Kasai H., Li Y. X., Miyashita Y. (1993). Subcellular-distribution of Ca^2+^ release channels underlying Ca^2+^ waves and oscillations in exocrine pancreas. Cell.

[11] Straub S. V., Giovannucci D. R., Yule D. I. (2000). Calcium wave propagation in pancreatic acinar cells: functional interaction of inositol 1,4,5-trisphosphate receptors, ryanodine receptors, and mitochondria. J. Gen. Physiol..

[12] Thorn P., Lawrie A. M., Smith P. M., Gallacher D. V., Petersen O. H. (1993). Local and global cytosolic Ca^2+^ oscillations in exocrine cells evoked by agonists and inositol trisphosphate. Cell.

[13] Yule D. I., Stuenkel E., Williams J. A. (1996). Intercellular calcium waves in rat-pancreatic acini: mechanism of transmission. Am. J. Physiol..

[14] Voronina S., Longbottom R., Sutton R., Petersen O. H., Tepikin A. (2002). Bile acids induce calcium signals in mouse pancreatic acinar cells: implications for bile-induced pancreatic pathology. J. Physiol..

[15] Petersen O. H., Tepikin A. V. (2008). Polarized calcium signaling in exocrine gland cells. Annu. Rev. Physiol..

[16] Feske S., Gwack Y., Prakriya M., Srikanth S., Puppel S. H., Tanasa B., Hogan P. G., Lewis R. S., Daly M., Rao A. (2006). A mutation in Orai1 causes immune deficiency by abrogating CRAC channel function. Nature.

[17] Liou J., Kim M. L., Heo W. D., Jones J. T., Myers J. W., Ferrell J. E., Meyer T. (2005). STIM is a Ca^2+^ sensor essential for Ca^2+^-store-depletion-triggered Ca^2+^ influx. Curr. Biol..

[18] Luik R. M., Wu M. M., Buchanan J., Lewis R. S. (2006). The elementary unit of store-operated Ca^2+^ entry: local activation of CRAC channels by STIM1 at ER–plasma membrane junctions. J. Cell Biol..

[19] Roos J., DiGregorio P. J., Yeromin A. V., Ohlsen K., Lioudyno M., Zhang S. Y., Safrina O., Kozak J. A., Wagner S. L., Cahalan M. D. (2005). STIM1, an essential and conserved component of store-operated Ca^2+^ channel function. J. Cell Biol..

[20] Hogan P. G., Lewis R. S., Rao A. (2010). Molecular basis of calcium signaling in lymphocytes: STIM and ORAI. Annu. Rev. Immunol..

[21] Parekh A. B., Putney J. W. (2005). Store-operated calcium channels. Physiol. Rev..

[22] Putney J. W. (2007). Recent breakthroughs in the molecular mechanism of capacitative calcium entry (with thoughts on how we got here). Cell Calcium.

[23] Lee K. P., Yuan J. P., Hong J. H., So I., Worley P. F., Muallem S. (2010). An endoplasmic reticulum/plasma membrane junction: STIM1/Orai1/TRPCs. FEBS Lett..

[24] Derler I., Schindl R., Fritsch R., Heftberger P., Riedl M. C., Begg M., House D., Romanin C. (2013). The action of selective CRAC channel blockers is affected by the Orai pore geometry. Cell Calcium.

[25] Rice L. V., Bax H. J., Russell L. J., Barrett V. J., Walton S. E., Deakin A. M., Thomson S. A., Lucas F., Solari R., House D., Begg M. (2013). Characterization of selective calcium-release activated calcium channel blockers in mast cells and T-cells from human, rat, mouse and guinea-pig preparations. Eur. J. Pharmacol..

[26] Sutton R., Criddle D., Raraty M. G., Tepikin A., Neoptolemos J. P., Petersen O. H. (2003). Signal transduction, calcium and acute pancreatitis. Pancreatology.

[27] Sutton R., Petersen O. H., Pandol S. J. (2008). Pancreatitis and calcium signalling: report of an international workshop. Pancreas.

[28] Tepikin A. V., Voronina S. G., Gallacher D. V., Petersen O. H. (1992). Acetylcholine-evoked increase in the cytoplasmic Ca^2+^ concentration and Ca^2+^ extrusion measured simultaneously in single-mouse pancreatic acinar-cells. J. Biol. Chem..

[29] Weber H., Huhns S., Luthen F., Jonas L. (2009). Calpain-mediated breakdown of cytoskeletal proteins contributes to cholecystokinin-induced damage of rat pancreatic acini. Int. J. Exp. Pathol..

[30] Voronina S. G., Sherwood M. W., Gerasimenko O. V., Petersen O. H., Tepikin A. V. (2007). Visualizing formation and dynamics of vacuoles in living cells using contrasting dextran-bound indicator: endocytic and nonendocytic vacuoles. Am. J. Physiol. Gastrointest. Liver Physiol..

[31] van Acker G. J., Perides G., Steer M. L. (2006). Co-localization hypothesis: a mechanism for the intrapancreatic activation of digestive enzymes during the early phases of acute pancreatitis. World J. Gastroenterol..

[32] Gerasimenko J. V., Gryshchenko O., Ferdek P. E., Stapleton E., Hebert T. O., Bychkova S., Peng S., Begg M., Gerasimenko O. V., Petersen O. H. (2013). Ca^2+^ release-activated Ca^2+^ channel blockade as a potential tool in antipancreatitis therapy. Proc. Natl. Acad. Sci. U.S.A..

