# Supplementary material for: The role of Ca2+ influx in endocytic vacuole formation in pancreatic acinar cells
Source: Biochem J. 2015 Jan 22;465(Pt 3):405–12. doi: 10.1042/BJ20140398 (PMC4303308; doi:10.1042/BJ20140398)
Supplement: Supplementary data [file bj4650405ntsadd.pdf]

Supplementary Material

The role of Ca^{2+} influx in endocytic vacuole formation in pancreatic acinar cells

Svetlana Voronina[1]*, David Collier[1*†], Michael Chvanov*, Ben Middlehurst*, Alison J. Beckett*, Ian A. Prior*, David N. Criddle*, Malcolm Begg[‡], Katsuhiko Mikoshiba[†], Robert Sutton[§] and Alexei V.Tepikin*.

[*]Department of Cellular and Molecular Physiology and [§] NIHR Liverpool Pancreas Biomedical Research Unit, the University of Liverpool, Crown Street, Liverpool L69 3BX, UK.
[†]Laboratory for Developmental Neurobiology, Riken Brain Science Institute, 2-1 Hirosawa, Wako City, Saitama, 351-0198 Japan.
[‡]Respiratory Therapy Area Unit, Medicines Research Centre, GlaxoSmithKline, Stevenage SG1 2NY, England, United Kingdom.

SUPPLEMENTARY FIGURES

Figure S1. GSK-7975A inhibits Ba^{2+} influx in unstimulated pancreatic acinar cells
(A) An example trace of Fura-2 fluorescence ratio changes recorded from an unstimulated acinar cell. Ba^{2+} slowly accumulated in the cell increasing the fluorescence ratio (representative of 72 cells). **(B)** 10µM GSK-7975A inhibited Ba^{2+} accumulation (representative of 92 cells; note the change of the slope upon GSK-7975A addition). **(C)** Proportional Ba^{2+} - induced change in the fluorescence ratio in the presence (added on 800 s, 92 cells) and absence (72 cells) of 10µM GSK-7975A. To calculate the proportional change (ΔBa^{2+}) the fluorescence ratio recorded at 800 s was subtracted from the fluorescence ratio recorded at 1700 s; the difference was then divided by the ratio recorded at 800 s and multiplied by 100%. GSK-7975A significantly, albeit incompletely, inhibited resting Ba^{2+} influx (which is considered as a reflection of resting Ca^{2+} influx).

Figure S2. Reversibility of the effects of GSK-7975A and PD150606 on the formation of endocytic vacuoles
The bar graph shows the numbers of vacuoles per cell, normalized to that formed as a result of CCK stimulation (second bar, 3 experiments). Approximately 30 cells were analyzed for each experiment described in this figure legend. 'Control' summarizes the results of measurements conducted on unstimulated cells (28 ± 6%, 3 experiments). The bar labels 'GSK-7975A+ Wash, CCK' summarizes the results of measurements in cells which were first treated with 10µM GSK-7975A (for 30 minutes), then washed by perfusion for approximately 5 minutes in standard extracellular solution (without agonist or inhibitor) and finally stimulated with CCK. The cells were maintained in CCK-containing solution for 60 minutes and number of vacuoles counted. The procedure involving preincubation with GSK-7975A followed by washing before CCK application resulted in the significant inhibition of endocytic vacuole formation (reduction to 48 ± 9%, 3 experiments). The reduction was slightly smaller than in the experiment shown in Fig.1 (when GSK-7975A was continuously

present), but the difference is not statistically significant. This experiment shows that the action of GSK-7975A is not easily reversible. The last bar of the graph summarises the results of experiments on cells that have been initially treated with PD150606, then washed by perfusion for approximately 5 minutes in standard extracellular solution (without agonist or inhibitor) and finally stimulated with CCK. The cells were maintained in CCK-containing solution for 60 minutes and the number of vacuoles counted. The number of vacuoles in this experiment (91 ± 20%, 3 experiments) was not significantly different from that in CCK stimulated cells (without inhibitor) indicating that the effect of PD150606 is reversible.

Figure S3. Reversibility of the effects of GSK-7975A on the CCK-induced Ca^{2+} plateau

(A) Partially reversible inhibition of the plateau component of the CCK-induced Ca^{2+} response by 10µM of GSK-7975A. Note the slow increase of Ca^{2+} following the GSK-7975A removal. Here and in (B) the example trace shows the normalized ratio of Fura-2 fluorescence recorded at excitation with 340nm and 380nm. The summary data for this type of experiment is shown in part (C). **(B)** Prolonged application of 10µM of GSK-7975 results in strong and sustained inhibition of the CCK-induced Ca^{2+} plateau. The summary data for this type of experiment is shown in part (C). **(C)** Bar graph summarizing the results of experiments illustrated in A and B shows the normalised amplitudes of the Ca^{2+} plateau (ΔCa^{2+}, calculated as the difference between the F340/F380 ratio at the end of experiment and just before the addition of CCK). The left part of the figure compares the normalised Ca^{2+} plateau amplitudes at 1500s. CCK-induced Ca^{2+} plateau (49 cells, an example trace for this type of measurements can be found in Figure 1A) and the CCK-induced Ca2+ plateau following treatment with 10µM of GSK-7975A (applied from 700s till 1500s, 104 cells) are significantly different. The mean value of CCK-induced Ca^{2+} plateau measured at 1500 s was used to normalise all measurements displayed on (C). The right part of the figure shows the Ca^{2+} plateau amplitudes measured at 3000s. The CCK- induced Ca^{2+} plateau did not substantially change between 1500s and 3000s. The next bar (hatched) summarises the Ca^{2+} plateau measurements in the cells that were first treated with 10µM of GSK-7975A (applied from 700s till 1500s) and then continuously perfused (i.e. washed) with GSK-7975A-free CCK-containing solution (104 cells). The right bar (filled) summarises the Ca^{2+} plateau measurements in the cells that were treated 10µM of GSK-7975A until 3000s (104 cells). The statistically significant differences in the amplitudes (compare the hatched bars labelled CCK, GSK-7975A (700s-1500s) for 1500s and 3000s graphs as well as bars labelled CCK, GSK-7975A (700s-1500s) and GSK-7975A (700s-3000s) for 3000 s graph) indicate the partial reversibility of GSK-7975A inhibition.

Figure S4. Formation of endocytic vacuoles in pancreatic acinar cells: effects of thapsigargin, bile acid and CCK

The bars show the numbers of vacuoles per cell, with the corresponding limits of standard errors, in control (unstimulated) cells and in cells stimulated for 1 hour by 1 µM thapsigargin (TG) or 500 pM CCK or 500 µM TLC-S. The corresponding numbers of cells in these experiments were 675 (Control), 155 (TG), 524 (CCK) and 462 (TLC-S). In all experiments Ca^{2+} concentration in the extracellular solution was 1mM. The number of vacuoles in control was significantly different from that formed in cells stimulated by TG. The number of vacuoles in cells stimulated by CCK was not significantly different from that in cells stimulated by TLC-S.

Figure S5. Effects of GSK-7975A and PD150606 on amylase secretion from pancreatic acinar cells

The graph shows the percentage of total amylase released into the external solution from unstimulated cells (0 CCK) or cells stimulated with the indicated concentrations of CCK in the presence or absence of GSK-7975A or PD150606 (5-9 experiments were conducted for each of the described conditions). The amylase secretion was measured for a 30-minute period. GSK-7975A reduced the mean values of amylase release for basal secretion and for all tested concentrations of CCK but the difference was small and reached statistical significance only for 500pM CCK. PD150606 did not significantly change basal secretion or secretion at any tested CCK concentration.

Figure S6. GSK-7975A and PD150606 protect against cell death

Pancreatic acinar cells were continuously maintained in the presence of propidium iodide. The fluorescence of this probe increases when the plasma membrane of the cells is compromised, reflecting cell death.

(A) The fluorescence of propidium iodide (PI) after 12.5 hours of no stimulation (control, 23 experiments) or stimulation by CCK 10 nM in the presence or absence of 10 µM of GSK-7975A or PD150606 (17 experiments). Both GSK-7975A and PD150606 significantly reduced PI responses induced by CCK. **(B)** The fluorescence of propidium iodide (PI) after 12.5 hours of no stimulation (control, 23 experiments) or stimulation by TLC-S 500 µM in the presence or absence of 10 µM of GSK-7975A or PD150606 (11 experiments). Both GSK-7975A and PD150606 significantly reduced PI responses induced by TLC-S.

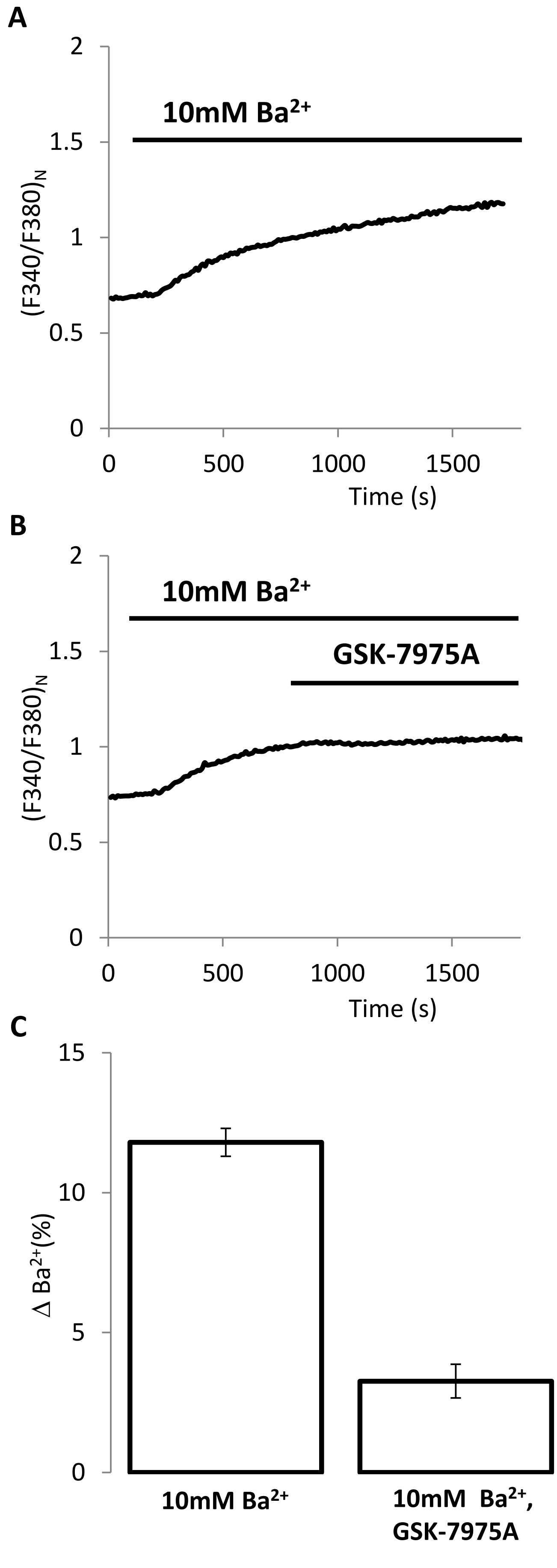
A
10mM Ba^{2+}
$(F340/F380)_N$
Time (s)
B
10mM Ba^{2+}
GSK-7975A
$(F340/F380)_N$
Time (s)
C
ΔBa^{2+}(%)
10mM Ba^{2+}
10mM Ba^{2+}, GSK-7975A

Fig S1

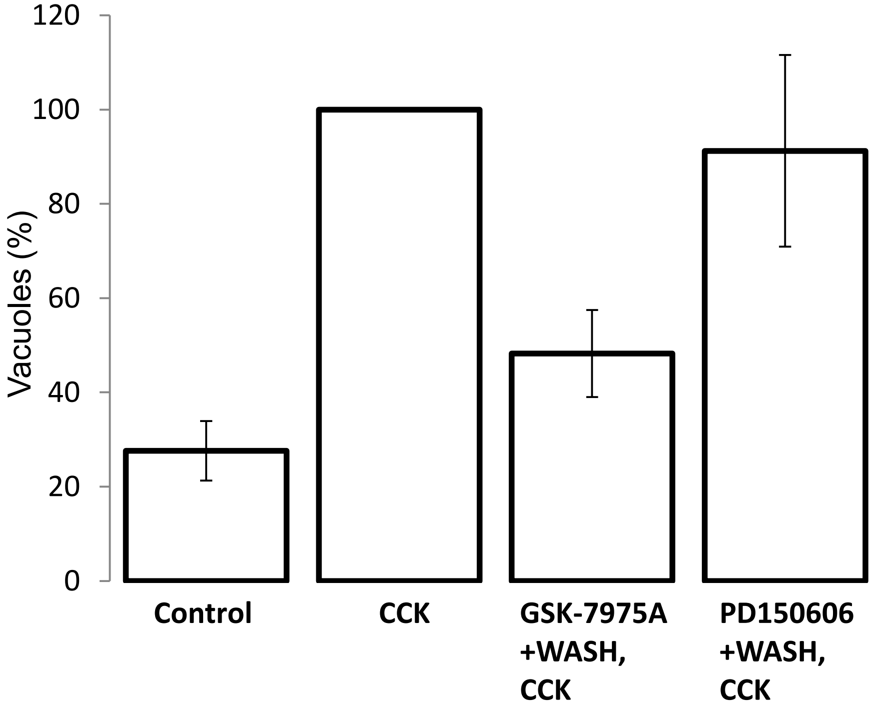

120
100
80
60
40
20
0
Vacuoles (%)
Control
CCK
GSK-7975A +WASH, CCK
PD150606 +WASH, CCK

Fig S2

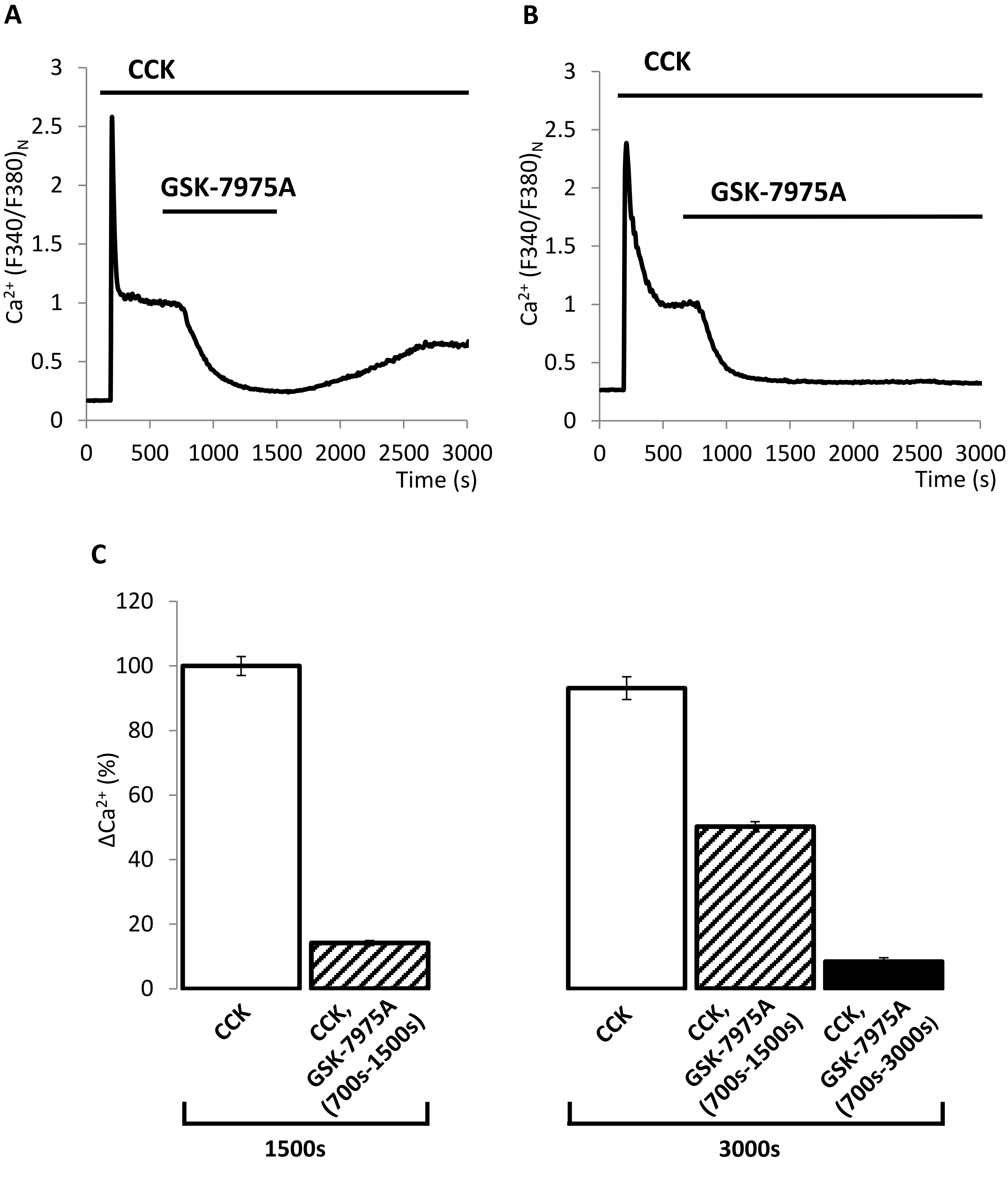
A
B
CCK
GSK-7975A
Ca^{2+} (F340/F380)$_N$
3
2.5
2
1.5
1
0.5
0
0
500
1000
1500
2000
2500
3000
Time (s)
C
120
100
80
60
40
20
0
ΔCa^{2+} (%)
CCK
CCK, GSK-7975A (700s-1500s)
CCK
CCK, GSK-7975A (700s-1500s)
CCK, GSK-7975A (700s-3000s)
1500s
3000s

Fig S3

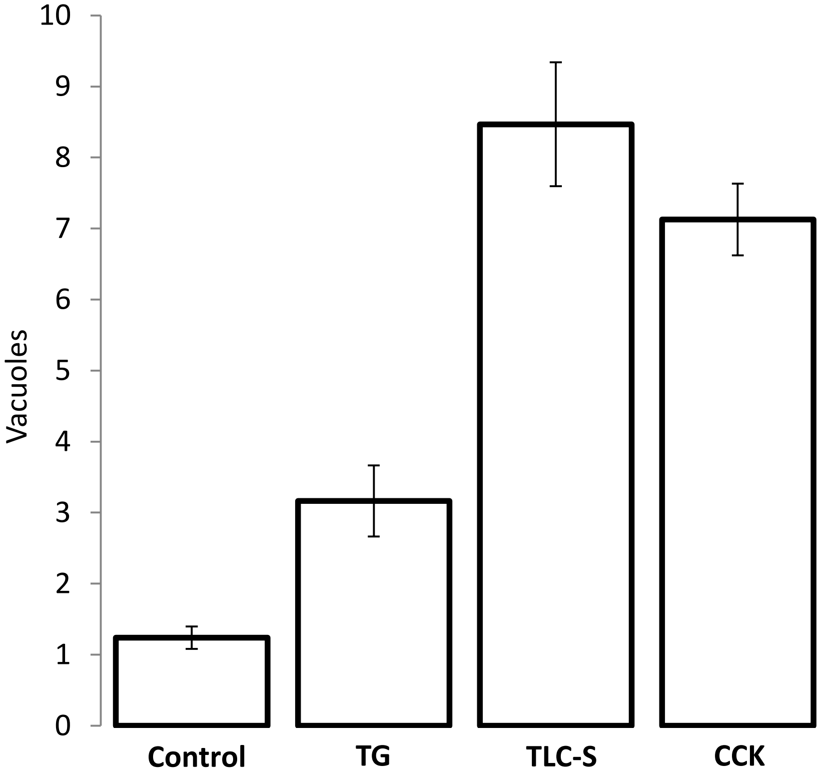

Fig S4

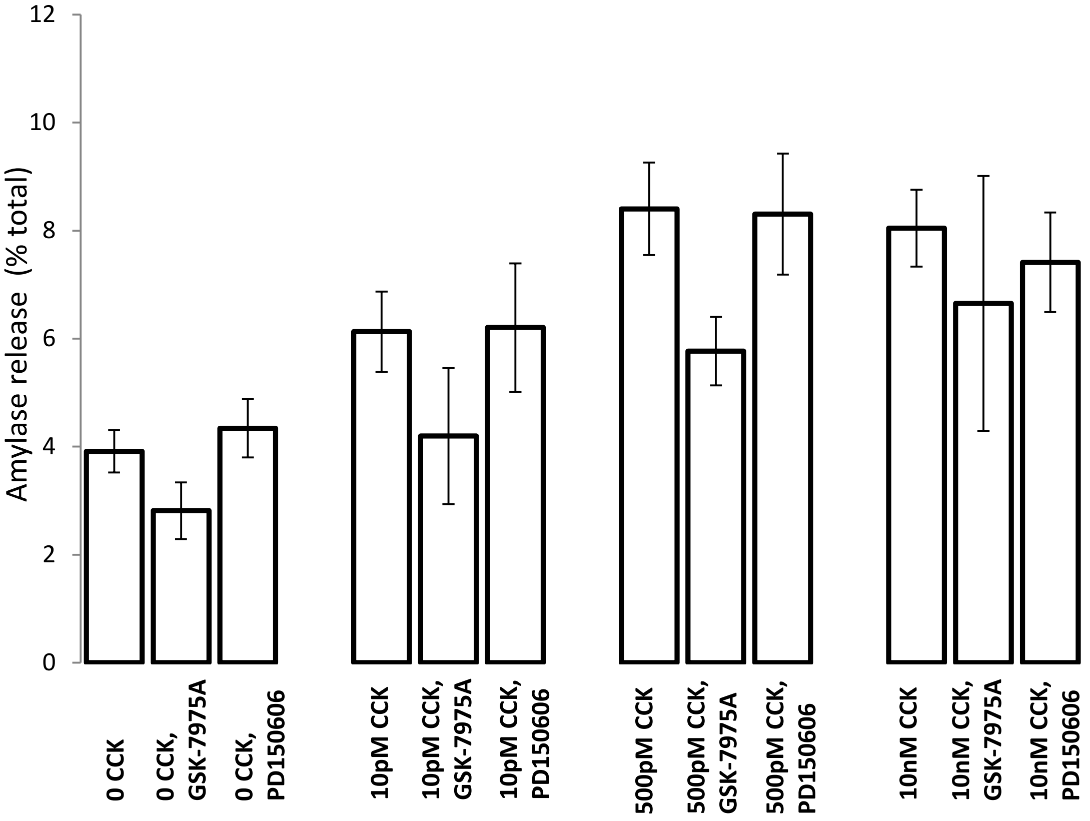

Amylase release (% total)
0
2
4
6
8
10
12
0 CCK
0 CCK, GSK-7975A
0 CCK, PD150606
10pM CCK
10pM CCK, GSK-7975A
10pM CCK, PD150606
500pM CCK
500pM CCK, GSK-7975A
500pM CCK, PD150606
10nM CCK
10nM CCK, GSK-7975A
10nM CCK, PD150606

Fig S5

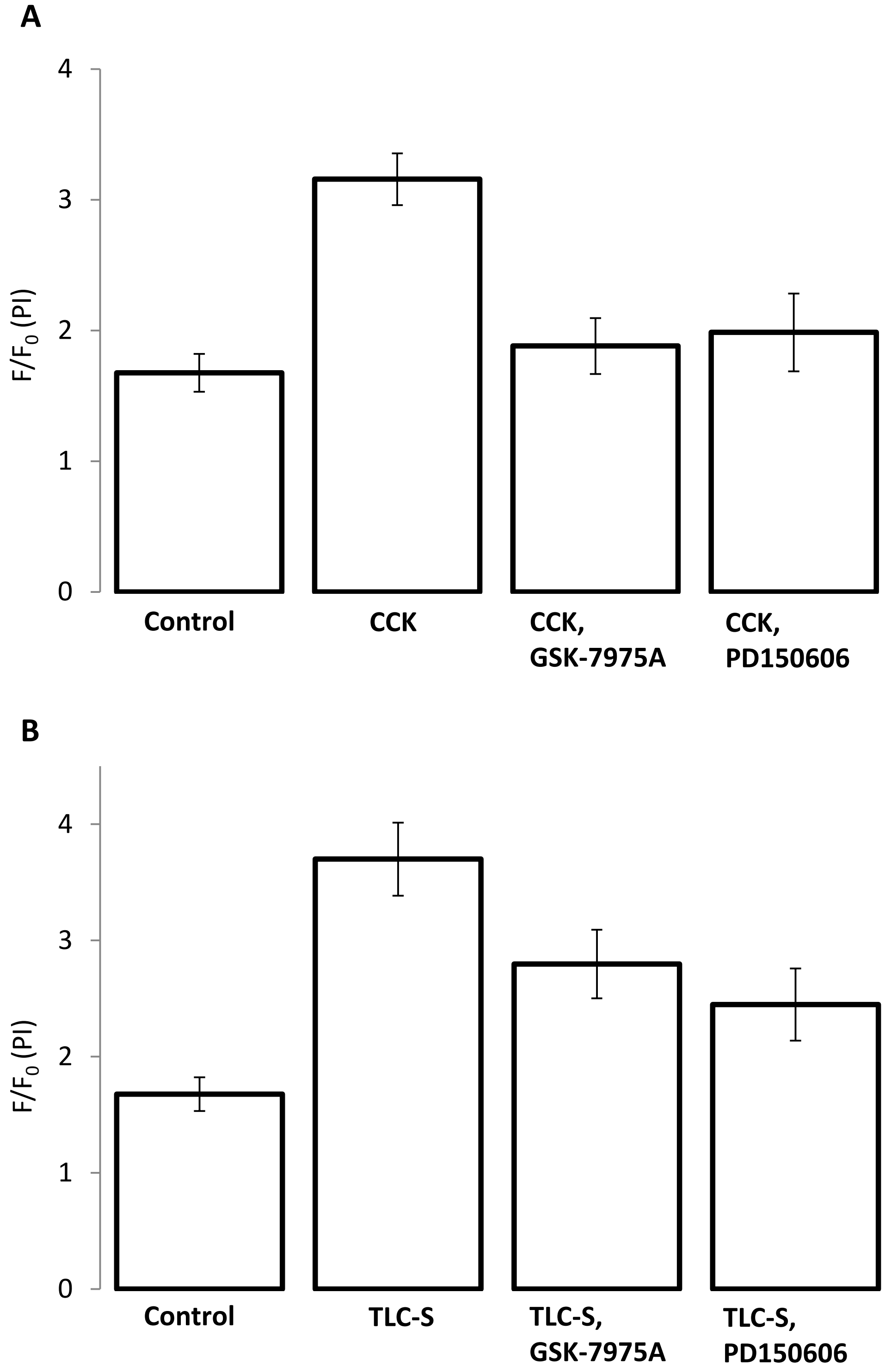
A
4
3
2
1
0
F/F0 (PI)
Control
CCK
CCK,
GSK-7975A
CCK,
PD150606
B
4
3
2
1
0
F/F0 (PI)
Control
TLC-S
TLC-S,
GSK-7975A
TLC-S,
PD150606

Fig S6
